# Causal associations of air pollution with rheumatoid arthritis: A transethnic Mendelian randomization study

**DOI:** 10.1371/journal.pone.0307514

**Published:** 2024-09-24

**Authors:** Ao He, Hainan Li, Shan Ouyang, Jia He, Zhuo Gong, Qingzhu Zhou, Songmei Wang, Xian Zhao

**Affiliations:** 1 Affiliated Calmette Hospital of Kunming Medical University, Plastic Surgery/Kunming First People’s Hospital, Plastic Surgery, Kunming, Yunnan, China; 2 Diqing Tibetan autonomous prefecture people’s hospital, Shangri-La, Yunnan, China; 3 School of Public Health, Kunming Medical University, Kunming, Yunnan, China; Chiang Mai University, THAILAND

## Abstract

**Background:**

Rheumatoid arthritis is a common rheumatic disease, and its onset is closely related to genetic and environmental factors, however, the relationship between air pollution and RA is still hotly debated. Further investigation of the relationship between air pollution and rheumatoid arthritis is conducive to a comprehensive understanding of the risk factors of the disease, providing certain value for the clinical prevention and treatment of RA.

**Methods:**

We used a Two-Sample Mendelian Randomization approach, integrating the large-scale public genomewide association study, to assess the genetically predicted causal effect of air pollution (including: PM2.5, PM2.5–10, PM10, nitrogen dioxide, nitrogen oxides) on RA in European and European East Asian populations, respectively. Indicators related to air pollution (2,505 individuals to 423,796 individuals), including European and East Asian populations were obtained from the Integrative Epidemiology Unit open GWAS project. Published East Asian RA data were also obtained from the IEU open GWAS project (212,453 individuals), while large-scale publicly available European RA data were obtained from finngen R10 (13,621 cases and 262,844 controls). Inverse variance weighting was used as the primary analytical method, complemented by MR-egger, Weighed median, and Weighted mode results. Cochran Q tested for heterogeneity, and MR-Egger regression analyses were performed to test for multiplicity. leave-one-out analysis allowed for the robustness and reliability were assessed.

**Results:**

No statistically significant effects of PM2.5, PM2.5–10, PM10, nitrogen dioxide, nitrogen oxides and RA were observed in either European or East Asian populations. Results from European data: PM2.5 (IVW OR: 0.71; 95% CI: 0.27–1.91; p = 0.498; number of SNPs: 5), PM2.5–10 (IVW OR: 1.20; 95% CI: 0.61–2.40; p = 0.596; number of SNPs: 15), PM10 (IVW OR: 1.69; 95% CI: 0.84–3.39; p = 0.142; number of SNPs: 9), nitrogen dioxide (IVW OR: 3.88; 95% CI: 0.19–77.77; p = 0.375; number of SNPs: 2), nitrogen oxides (IVW OR: 0.51; 95% CI: 0.16–1.67; p = 0.268; number of SNPs: 4). East Asian data results: PM2.5 (IVW OR: 1.16; 95% CI: 0.98–1.38; p = 0.086; number of SNPs: 4), PM2.5–10 (IVW OR: 1.14; 95% CI: 0.95–1.38; p = 0.166; number of SNPs: 2), PM10 (IVW OR: 0.95; 95% CI: 0.81–1.11; p = 0.503; number of SNPs: 3), nitrogen dioxide (IVW OR: 0.87; 95% CI: 0.76–1.00; p = 0.051; number of SNPs: 6), nitrogen oxides (IVW OR: 0.96; 95% CI: 0.82–1.14; p = 0.671; number of SNPs: 3). No signs of pleiotropy or heterogeneity were observed in the MR-Egger intercept, MR-PRESSO and Cochrane’s Q (p>0.05). In addition, no outliers were found in the MR-PRESSO analysis. The results were further validated by leave-one-out tests, confirming the robustness of the findings.

**Conclusions:**

We performed transethnic MR analysis suggesting that there may not be a genetically predicted causal relationship between air pollution and RA.

## Introduction

A type of chronic joint inflammation, rheumatoid arthritis (RA) mainly results from the immune system erroneously targeting the body’s tissues, causing bone loss by stimulating bone breakdown and suppressing bone formation. It usually affects the small joints of the hands and feet [1]. At the same time, systemic complications, such as heart disease, bone loss, lung problems and infection risk, are linked to RA. It significantly affects the quality of life of patients, leading to physical limitations, emotional distress and a decreased sense of overall well-being [2]. In the U.S. population, the prevalence of RA is about 1%. And the prevalence in North Africa, the Middle East, and Asia are about 0.16 percent [3]. Social determinants of health (SDOH), like health care access and use, and income level, may explain these differences [4]. Also, Genetic and environmental factors interact to cause RA, a disease with multiple factors [5]. In recent years, Potential risk factors for RA development include industrialization and the resulting air pollution. The relationship between the two has sparked intense debate.

In recent years, as global industrialization has progressed, the ensuing air pollution has become an urgent problem for countries to address. Air pollution is known to cause damage to the human body through various pathways, and inflammation and oxidative stress are recognized as common underlying mechanisms of damage caused by air pollution. Air pollutants, especially fine particulate matter (PM2.5) and ultrafine particulate matter (PM0.1), ozone, nitrogen oxides, and transition metals, are strong oxidizers or capable of generating ROS, and the relationship between these particulate matter and gaseous pollutants, which have emerged as potential risk factors for the development of rheumatoid arthritis (RA), has been hotly debated. However, potential confounders and reverse causality may bias the results of traditional observational studies, while experimental studies are challenged by exposure to air pollution of varying concentrations and durations, small sample sizes, and other factors that have also hampered the demonstration of a relationship [6–9].

In epidemiological research, Mendelian randomization (MR) is commonly employed to assess the genetically predicted causal relationship between exposures and outcomes using genetic variation as an instrumental variable. The fundamental principle of MR lies in the random mutation of genetic information transmitted to the next generation upon conception, ensuring that they are not subject to genetic confounding. By minimizing potential confounders and reducing the effects of reverse causation [10, 11], MR has become a practical alternative to randomized controlled trials (RCTs), especially where cost and feasibility constraints limit implementation. The aim of this study was to investigate the genetically predicted association of air pollutants (PM2.5, PM2.5–10, PM10, nitrogen dioxide, and nitrogen oxides) with RA, considering the genetic differences between European and East Asian populations. For this purpose, we performed genome-wide association analysis (GWAS) in different population subgroups [12]. The analysis at the genetic level will provide new insights into the clinical treatment and mitigation of RA, as well as providing a reliable reference for the formulation of air pollution treatment and management tactics.

## Materials and methods

### Sources of data

#### Exposure datas

In this research, we utilized various air pollution-associated characteristics, encompassing particulate matter PM2.5, PM2.5–10, PM10, nitrogen dioxide, and nitrogen oxides, as determinants of exposure. SNPs linked to these exposures were specifically selected from the IEU OpenGWAS initiative (https://gwas.mrcieu.ac.uk/datasets/) for this study. They involved 423,796 to 456,380 participants of European origin and 2,505 to 2,625 participants of East Asian origin, respectively (Table 1).

**Table 1:** Detailed information of data sources.

Phenotypes	Ref/IEU ID	PMID	Consortium	Ancestry	Participants	Sex	Year
Particulate matter air pollution (pm2.5)	ukb-b-10817	30504882	UKB	European	423,796 individuals	Males and Females	2018
Particulate matter air pollution 2.5-10um	ukb-b-12963	30504882	UKB	European	423,796 individuals	Males and Females	2018
Particulate matter air pollution (pm10)	ukb-b-589	30504882	UKB	European	455,314 individuals	Males and Females	2018
Nitrogen dioxide air pollution	ukb-b-2618	30504882	UKB	European	456,380 individuals	Males and Females	2018
Nitrogen oxides air pollution	ukb-b-12417	30504882	UKB	European	456,380 individuals	Males and Females	2018
Particulate matter air pollution (pm2.5)	ukb-e-24006_EAS	30504882	UKB	East Asian	2,505 individuals	Males and Females	2020
Particulate matter air pollution 2.5-10um	ukb-e-24008_EAS	30504882	UKB	East Asian	2,505 individuals	Males and Females	2020
Particulate matter air pollution (pm10)	ukb-e-24005_EAS	30504882	UKB	East Asian	2,505 individuals	Males and Females	2020
Nitrogen dioxide air pollution	ukb-e-24016_EAS	30504882	UKB	East Asian	2,625 individuals	Males and Females	2020
Nitrogen oxides air pollution	ukb-e-24004_EAS	30504882	UKB	East Asian	2,625 individuals	Males and Females	2020
Rheumatoid arthritis	M13_RHEUMA	36653562	Finngen	European	13,621 cases and 262,844 controls	Males and Females	2023
Rheumatoid arthritis	bbj-a-151	34594039	BioBank Japan	East Asian	4,199 cases and 208,254 controls	Males and Females	2019

Note: BBJ: Biobank Japan; UKB: UK Biobank; TSMR: Two-Sample Mendelian Randomization.

#### Outcome datas

For the outcome dataset, GWAS data for RA in East Asian populations were also obtained from the IEU open GWAS, with 4,199 cases and 208,254 controls. GWAS data for RA in European populations were obtained from Finngen R10 studies (https://r10.finngen.fi/) with 13,621 cases and 262,844 controls. The GWAS on exposure and outcome had no sample overlap, which is important to note (Table 1).

### Study design

**Fig 1:**
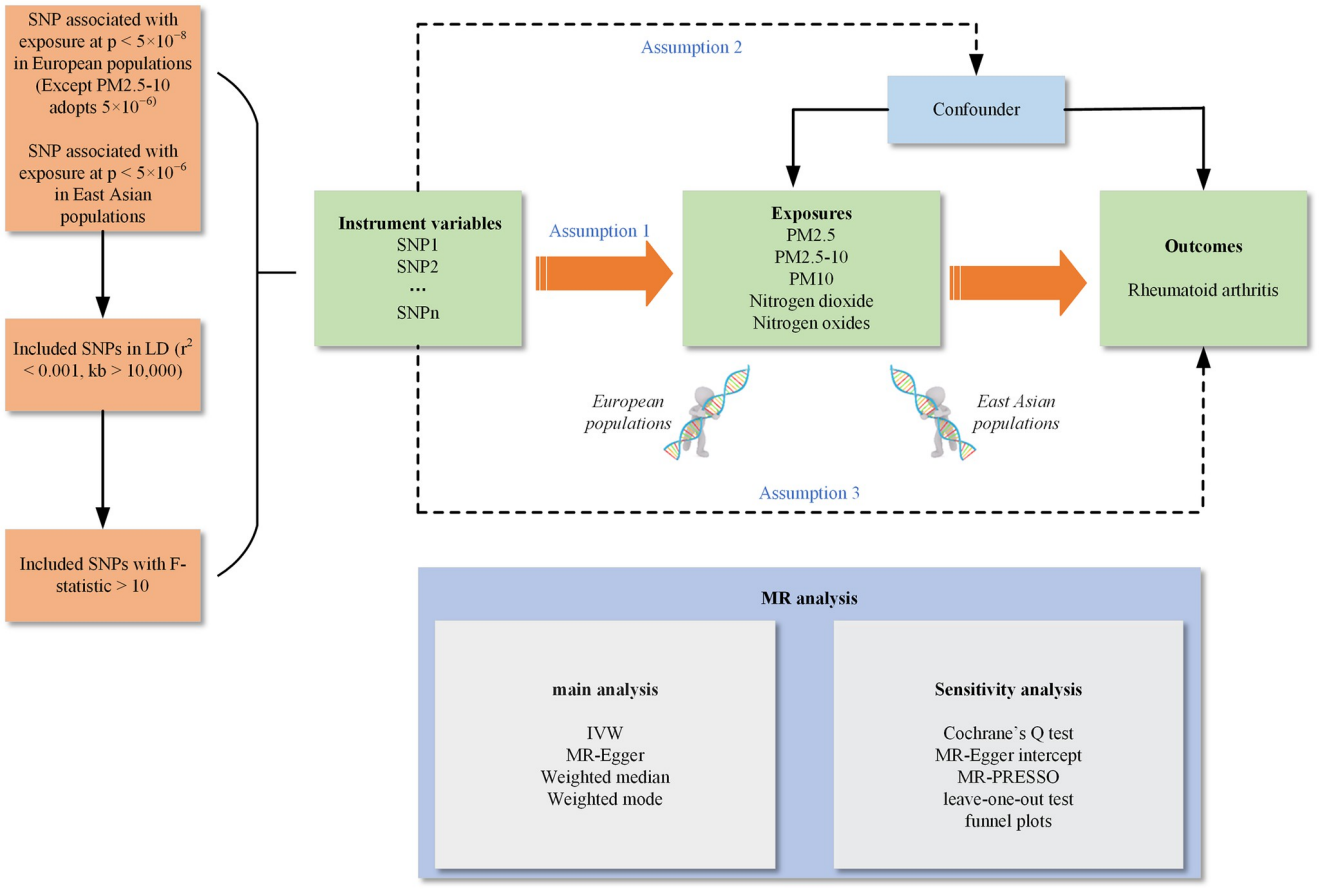
Flowchart of Mendelian randomization analyses conducted in this study. LD, linkage disequilibrium; r^2^, linkage disequilibrium coefficient; kb, linkage disequilibrium block size; SNPs, Single nucleotide polymorphisms; Assumption 1, Correlation hypothesis: There is a strong correlation between SNPs and exposure factors; Assumption 2, Independence assumption: SNPs and confounding factors are independent; Assumption 3, Exclusion hypothesis: SNPs can only affect outcome through exposure factors; MR, Mendelian randomization; IVW, Inverse variance weighted; MR-PRESSO, MR Pleiotropy RESidual Sum and Outlier.

Instrumental variables (IVs) are employed to represent single-nucleotide polymorphisms (SNPs) in this study [13]. The MR design follows three key assumptions: (1) It is crucial for the IVs to exhibit a robust correlation with the exposure factors, which encompass PM2.5, PM2.5–10, PM10, nitrogen dioxide, and nitrogen oxides; (2) IVs should lack associations with confounding factors; (3) IVs should exclusively influence RA through air pollution factors without genetic pleiotropy [14, 15]. Our research design is presented using a flowchart (Fig 1). In order to extract a sufficient number of SNPs, we set the significance level of the data for PM2.5–10 in the European population and all Asian populations to (p < 5 × 10^−6^, kb > 10,000 and r^2^ < 0.001). For PM2.5, PM10, nitrogen dioxide, nitrogen oxides in the remaining European populations, we set the significance level to (p < 5 × 10^−8^, kb > 10,000 and r^2^ < 0.001) [16]. In addition, Exposure and IVs should have a strong correlation, with F-statistic values above 10 as the measure of strength; otherwise they were considered weak instrumental variables and were excluded [12, 17]. We searched the Phenoscanner website to remove confounders related to air pollution and RA, including some confounders associated with cell count of white blood, BMI, alcohol consumption, and smoking, to avoid potential confounding affecting the results [18] (S1 Table in S1 File). To ensure that causality was in the right direction, we implemented MR-Steiger filtering to remove SNPs that had a strong correlation with the outcome [19, 20]. To correctly estimate the effect of exposure and outcome, it is essential that the same allele is always linked to the SNPs. One of the most important criteria for SNPs is that they were not palindromic SNPs (A/T or C/G) with intermediated EAFs (40–70%), we excluded these palindromic SNPs because they may produce higher false-positive results due to genotyping errors.

### MR analysis

To assess the impact of air pollution-induced characteristics on RA, we employed the inverse variance weighted (IVW) technique as the primary analysis method in European and East Asian populations. By combining the Wald values for each SNP, meta-analysis methods are employed by IVW to acquire a comprehensive estimate of the impact of exposure on outcomes. Assuming that all SNPs are valid instruments, IVW provides the most accurate estimates of causal effects. In addition, to improve reliability, we conducted supplementary analyses using MR-Egger, Weighted median (WM) and Weighted mode methods. MR-Egger regression analyses showed robustness to invalid instruments and addressed horizontal pleiotropy by introducing parameters that accounted for potential bias. The WM approach collects up to 50% of the analyzed information from genetic variation in intrinsic to zero IVs [21–23]. In order to ascertain the dependability and strength of the primary findings, we conducted numerous sensitivity analyses. (1) MR-PRESSO outlier test was applied to identify and correct for pleiotropy and outliers. And a p-value below 0.05 for the MR-Egger intercept indicated horizontal pleiotropy [23, 24]. (2) Cochran’s Q test is used to identify heterogeneity due to horizontal pleiotropy and other biases. A p-value below 0.05 for Cochran’s Q test indicates the existence of heterogeneity. To tackle heterogeneity, the IVW method employed the random effects model, whereas the fixed effects model of the IVW method was adopted as an alternative approach in this case [25]. (3) Leave-one-out analysis was employed to assess the stability of effect sizes and identify SNPs with disproportionate influence on the association. In this process, each SNP was systematically excluded one at a time, and the IVW method was then applied to the remaining SNPs [23].

## Results

### Main analysis results for MR

**Fig 2:**
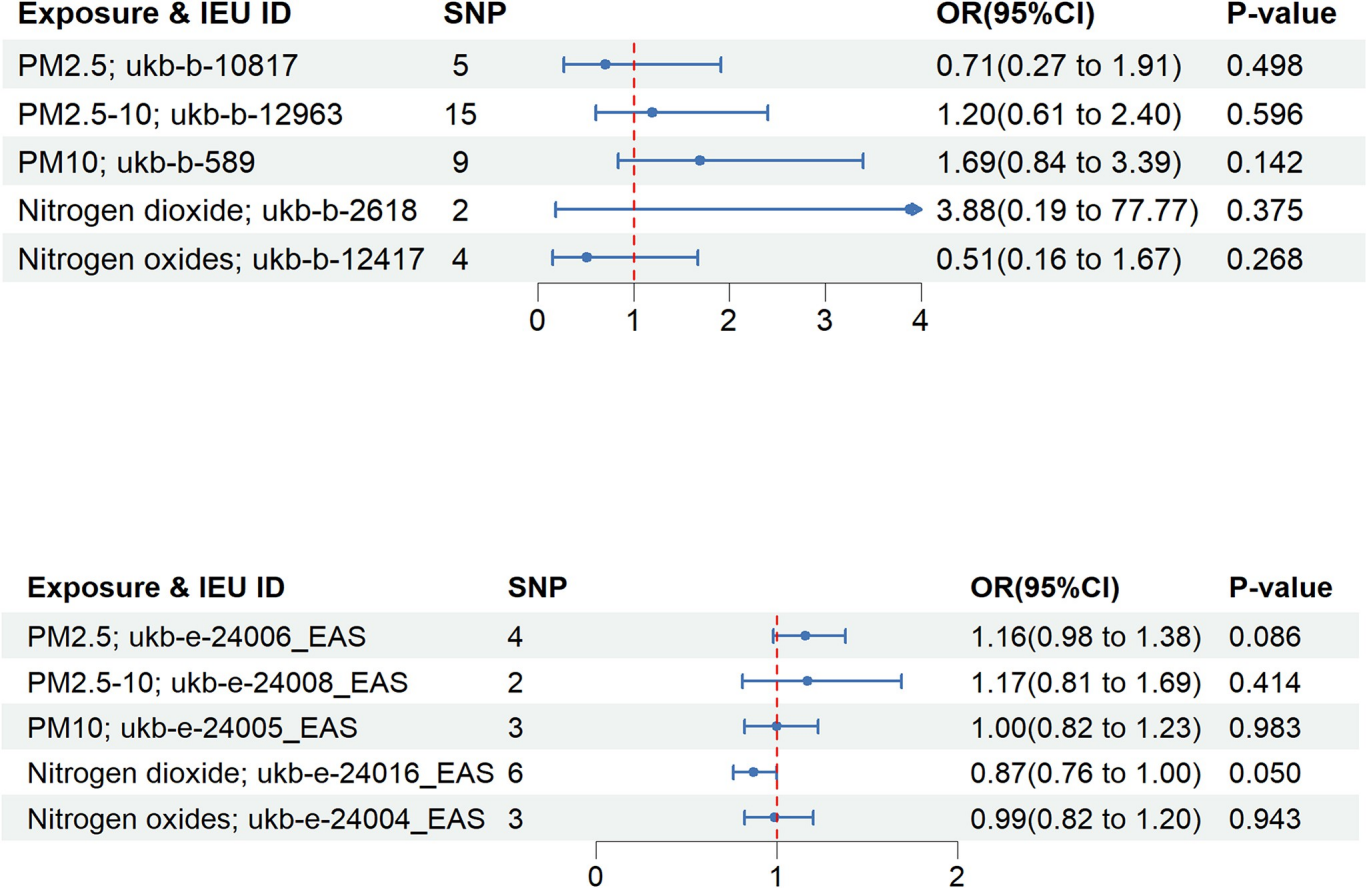
The forest plot displays the associations of genetically predicted levels of PM2.5, PM2.5–10, PM10, nitrogen dioxide, and nitrogen oxide with the risk of rheumatoid arthritis. SNP: Single-nucleotide polymorphism; CI: confidence interval; OR: odds ratio. P-value: P-value of the IVW method.

This study strictly adhered to the three major hypotheses of MR. After excluding single nucleotide polymorphisms (SNPs) that displayed strong associations with confounding variables and the outcome, and performing the elimination of the palindromic structure, we extracted 2–15 SNPs from European populations and 3–6 SNPs from East Asian populations for the subsequent analyses. F-statistic values ranged from 23.37–34.32 in European populations and 21.09–25.83 in East Asian populations, without the presence of weak instrumental variables. With explained variances ranging from 0.02% to 0.08% in European populations and from 2.04% to 4.79% in East Asian populations (S2 and S3 Tables in S1 File). Since IVW provides the most accurate estimates of causal effects, we assessed significant genetically predicted causal relationships primarily based on IVW p-values. In the MR population stratification analysis, we did not identify a significant genetically predicted causal association between air pollution and RA, and the results of the main analyses are presented using forest plots (Fig 2). Results from European data: PM2.5 (p = 0.498; IVW OR: 0.71; 95% CI: 0.27–1.91), PM2.5–10 (p = 0.596; IVW OR: 1.20; 95% CI: 0.61–2.40), PM10 (p = 0.142; IVW OR: 1.69; 95% CI: 0.84–3.39), nitrogen dioxide (p = 0.375; IVW OR: 3.88; 95% CI: 0.19–77.77), nitrogen oxides (p = 0.268; IVW OR: 0.51; 95% CI: 0.16–1.67). East Asian data results: PM2.5 (p = 0.086; IVW OR: 1.16; 95% CI: 0.98–1.38), PM2.5–10 (p = 0.166; IVW OR: 1.14; 95% CI: 0.95–1.38), PM10 (p = 0.503; IVW OR: 0.95; 95% CI: 0.81–1.11), nitrogen dioxide (p = 0.051; IVW OR: 0.87; 95% CI: 0.76–1.00), nitrogen oxides (p = 0.671; IVW OR: 0.96; 95% CI: 0.82–1.14). In addition, MR-Egger, WM, and the Weighted mode did not reveal significant causality.

### Heterogeneity and sensitivity analysis

In order to make our conclusions robust and reliable, we performed heterogeneity tests and sensitivity analyses. Cochran’s Q-test indicated the absence of heterogeneity in the main results. (p > 0.05), whereas The MR-Egger intercept found no signs of potential horizontal pleiotropy (p > 0.05). Similarly, the MR-PRESSO outlier test did not identify any outliers. In addition, all SNPs included in the analysis passed the Steiger test. The stability of the results was confirmed through the leave-one-out test. The causality results between air pollution and RA obtained from our population stratification are demonstrated to be reliable (Table 2). Because only two SNPs were extracted for the two exposures, nitrogen dioxide from European population sources and PM2.5–10 from East Asian population sources could not perform sensitivity analyses. S1–S4 Figs displayed leave-one-out test and scatter plots of MR analyses conducted for European and East Asian populations.

**Table 2:** MR sensitivity analysis.

**Effect of air pollution on rheumatoid arthritis (European populations)**												
**Exposure**	**Outcome**	**SNPs**	**MR-Egger intercept**			**MR-PRESSO**		**Cochrane’s Q**			**Steiger_test**	
			**Intercept**	**SE**	***Pval***	***P*-value**	***Outlier***	***Q***	***Q_df***	***Q_pval***	**Direction**	***Pval***
Particulate matter air pollution (pm10)	Rheumatoid arthritis	9	-0.033	0.910	0.068	0.121	NA	12.702	8	0.123	TRUE	8.65E-15
Nitrogen dioxide air pollution	Rheumatoid arthritis	2	NA	NA	NA	NA	NA	NA	NA	NA	TRUE	1.30E-03
Particulate matter air pollution (pm2.5)	Rheumatoid arthritis	5	0.015	1.794	0.513	0.607	NA	1.869	4	0.760	TRUE	2.64E-11
Nitrogen oxides air pollution	Rheumatoid arthritis	4	-0.097	4.046	0.305	0.261	NA	3.698	3	0.296	TRUE	3.41E-07
Particulate matter air pollution 2.5-10um	Rheumatoid arthritis	15	-0.013	0.931	0.400	0.470	NA	18.209	14	0.197	TRUE	3.71E-17
**Effect of air pollution on rheumatoid arthritis (East Asian populations)**												
**Exposure**	**Outcome**	**SNPs**	**MR-Egger intercept**			**MR-PRESSO**		**Cochrane’s Q**			**Steiger_test**	
			**Intercept**	**SE**	***Pval***	***pval***	***Outlier***	***Q***	***Q_df***	***Q_pval***	**Direction**	***Pval***
Particulate matter air pollution (pm10)	Rheumatoid arthritis	3	-0.036	2.042	0.920	0.920	NA	3.703	2	0.157	TRUE	2.02E-15
Nitrogen dioxide air pollution	Rheumatoid arthritis	6	0.024	0.439	0.556	0.755	NA	3.265	5	0.659	TRUE	2.82E-28
Particulate matter air pollution (pm2.5)	Rheumatoid arthritis	4	0.007	0.216	0.653	0.868	NA	2.101	3	0.552	TRUE	8.92E-20
Nitrogen oxides air pollution	Rheumatoid arthritis	3	0.001	3.174	0.997	0.998	NA	1.407	2	0.495	TRUE	1.15E-16
Particulate matter air pollution 2.5-10um	Rheumatoid arthritis	2	NA	NA	NA	NA	NA	NA	NA	NA	TRUE	4.53E-12

Note: SE: standard error. MR-PRESSO: Mendelian Randomization Pleiotropy RESidual Sum and Outlier method.

## Discussion

A type of chronic systemic autoimmune disease, RA is characterized by severe and symmetric inflammation of the joints and other organs, which is incurable because the cause is still unknown. In order to explore and understand the pathogenesis of RA and to find better treatment modalities, many studies have focused on the etiologic and predisposing factors that cause the disease. It has been found that DNA repair defects leading to loss of T-cell tolerance and the emergence of aggressive T cells, or autoantibodies such as T cells and their mediated autoimmune responses, as well as cytokines may contribute to RA [26, 27]. BeiXu, JinLin et al. explored the pathogenesis of RA by collecting and examining 8,789 patients aged between 20 and 55 years with RA from the National Health and Nutrition Examination Survey (NHANES) database, and found that factors such as obesity, osteoporosis, advanced age, regular smoking, and low standard of living were all related to RA [28]. Understanding the mechanism of RA occurrence, reducing the risk factors and preventing the disease are more important. Air pollution is an unavoidable topic in various fields nowadays, and the research on the genetically predicted causal relationship of air pollution with RA is a hot research topic at present.

The risk of RA was assessed in an observational study of residents exposed to four levels of PM2.5 and NO_2_. The study showed that PM, nitrogen oxides, and other air pollutants induce inflammatory mediators and autoantibodies, which increased the susceptibility and severity of RA [29]. By analyzing the readmission rate of RA in Hefei from 2014 to 2018, another study found that high PM2.5 and NO_2_ exposure was connected to the odds of readmission increased for RA. This article acknowledges that women and the elderly were the main populations for RA readmissions [8]. By reviewing a nationwide dataset of 363 men and women, Giovanni Adami et al. reported that long-term air pollution exposure increased the likelihood of autoimmune diseases, including RA, connective tissue disease (CTD), and inflammatory bowel disease (IBD) [30]. Over eight years, JieZhang and colleagues tracked 342,973 people for RA onset and found that chronic exposure to air pollution could increase RA risk, especially in populations with higher genetic risk [31]. These studies, while they have tried to minimize confounding effects, still have the inherent limitations of retrospective studies. The same meta-analysis that provided quantitative evidence failed to demonstrate that PM exposure appears to have a negative effect on RA incidence. In the Stockholm area, air pollution and RA risk were not consistently or overall associated, as shown by another case-control study [32, 33]. The relationship between RA-associated autoantibodies and exposure to PM in patients without RA was evaluated in an experimental study, which concluded that RA-associated autoantibodies and joint tenderness or swelling were not associated with ambient PM concentrations [34]. Most previous epidemiologic studies were case-control designs that failed to elucidate causality, had vague chronology, and had many uncontrolled residual confounders. Even in prospective observational studies, there are problems of lost visit bias or small population sample sizes [35, 36]. Therefore, whether air pollution directly generates RA cannot be established by observational studies alone.

Through MR, the genetically predicted causal relationship between air pollution and RA was described by this article without precedent at the genetic level. Unsafe PM2.5 levels expose 7.3 billion people globally, with 80% from countries with lower per capita income [37]. Given the differences in air pollution across regions, we have stratified the population to make the results more generalizable [38]. We also used the most up-to-date and comprehensive GWAS data and overcomes many of the biases associated with traditional observational studies [31]. Our study can mimic an RCT in an observational setting. RCTs are the gold standard for causality studies, but they are costly and often unfeasible. However, MR studies simulate randomized groupings to some extent and can effectively avoid confounding bias in the random assignment of SNPs at the time of conception [11]. The overall results did not provide direct evidence regarding the causal link between the genetic predisposition to five phenotypes of air pollution and the risk of RA; thus, we infer that PM2.5, PM2.5–10, PM10, nitrogen dioxide, and nitrogen oxides have no direct genetically predicted causal relationship on RA. Nonetheless, we acknowledge the findings from prior studies that show a significant link between air pollution and RA, and we hypothesize that this effect is indirectly mediated by multiple factors. For example, gaseous pollutants may absorb UVB radiation and decrease serum vitamin D, thereby increasing the incidence of RA [38]. At the same time, People may stay indoors more in areas with high air pollution levels and experience increased exposure to volatiles, low sulfur compounds, heavy metals, indoor dust, or tobacco [39]. Smoking and smoking-induced anticitrullinated protein antibody positivity increase the risk and severity of RA [40]. Long-term home use may produce chronic psychosocial stress and altered physical activity, which may indirectly affect the risk of RA [41]. This implies that RA etiology involves multiple factors.

This MR study offers a deeper and broader investigation of the causal link between air pollution and RA to some extent. However, our study has some limitations. First, limited by the current GWAS database, we obtained a small number of IVs, including two exposures for which only two strongly correlated IVs were extracted, and none of the analytical methods other than IVW were appropriate, so we were unable to identify potential heterogeneity and pleiotropy through sensitivity analysis. Second, the present study only involved participants from Europe and East Asia, so the applicability of the results to other populations is unclear. Therefore, future research on the causal link between air pollution and RA needs to incorporate samples from various ethnic backgrounds to enhance the generalizability of the results. Moreover, it is essential to examine the heterogeneity of RA patients, as different subtypes of RA patients may have distinct causal associations with air pollution. In view of the existing GWAS database resources for air pollution, this provides a genetic basis for Mendelian randomization analysis. In fact, a key aspect of Mendelian randomization (MR) research is the principle of gene-environment equivalence. As highlighted by professor George Davey Smith, this principle requires that the influence of genes on exposure variables should be equivalent to the influence of the environment in different situations. We chose air pollution (PM2.5) as the exposure variable based on the following reasons and evidence: (1) Biological correlation between genetic variants and air pollution: Studies have shown that certain genetic variants can affect an individual’s sensitivity to air pollution. These variants are directly linked to inflammatory pathways triggered by air pollution. For example, studies have found that in children, PM2.5 exposure interacts with methylation of iNOS gene promoters and NOS2 promoter haplotypes, affecting levels of airway inflammation [42]. (2) PM2.5-related GWAS studies: Existing studies using data from large-scale genome-wide association studies (GWAS) have identified genetic variants associated with air pollution exposure. These variables are statistically significant and meet the basic requirements of instrumental variables in MR Research. For example, a study using UK Biobank data identified several SNPs associated with PM2.5 exposure that could be used as instrumental variables in MR Studies to assess the causal effects of air pollution on autoimmune diseases. (3) In MR Studies, genetic variants selected as instrumental variables should be reasonably representative of environmental exposure and produce biologically similar effects to environmental exposure. The GWAS data for subjects in the air pollutant area in this paper were obtained from the UK Biobank, which was obtained from the MRC IEU (https://gwas.mrcieu.ac.uk/). As part of the European Air Pollution Effects Cohort Study (ESCAPE), the estimated annual mean of air pollution was derived from a Land use regression (LUR) model to estimate exposure by modelling the x-y coordinates of the baseline residential areas of each UK Biobank participant. While genetic variants are not the direct cause of PM2.5 exposure, they can serve as a useful tool for assessing the health effects of PM2.5 [43–45], especially when considering an individual’s sensitivity and reactivity to PM2.5. These studies used MR Design to explore the relationship between environmental exposures, including air pollution, and health outcomes, further supporting the rationale for including air pollution in the MR Framework. We will continue to explore the complex relationship between genetic variation and PM2.5 exposure and further validate these associations in future studies." We recognize the need for caution in MR studies, especially when using environmental exposure as a variable, to avoid "noodle" studies. Therefore, we ensured the validity and rigor of this study through rigorous IV selection and sensitivity analysis, as well as biological reasonableness. It is noteworthy that in the discourse on the link between air pollution and disease, the interplay between genetics and environment is often overlooked [46]. Studies focusing on the impact of ozone exposure have corroborated that polymorphisms in oxidative stress genes, such as NQO1, GSTM1, and GSTP1, can precipitate respiratory symptoms and elevate the risk of asthma development [47, 48]. However, C57BL/6 (TLR4-deficient) mice exhibit a protective phenotype, demonstrating no airway hyperresponsiveness following subchronic ozone exposure. Research involving genetically diverse mouse strains has identified specific DNA sequences on chromosomes 4, 11, and 17, encompassing Toll-like receptor 4 (TLR4) and numerous inflammatory cytokines, which are associated with increased pulmonary injury and inflammation induced by ozone [49]. This underscores the fact that genetic variation can significantly influence an individual’s susceptibility to air pollutants through various mechanisms involving antioxidant pathways [50, 51]. The combination of genetic susceptibility and the persistent stress of air pollution may heighten the risk of adverse health outcomes. For instance, air pollutants can activate stress-responsive regions in the central nervous system, such as the hypothalamic-pituitary-adrenal (HPA) axis, which has broad implications for immune system health and can increase susceptibility to immune-mediated diseases. Understanding these genetic factors is instrumental in identifying vulnerable populations and devising targeted interventions aimed at mitigating the health impact of air pollution.

## Conclusion

Our cross-ethnic Mendelian randomization analysis in this study showed no significant causal effect between genetically predicted air pollutants (PM2.5, PM2.5–10, PM10, nitrogen dioxide, and nitrogen oxides) and the incidence of rheumatoid arthritis in European and East Asian populations. To confirm the validity of our findings, we will conduct further research on the detrimental effects of air pollution on RA if more updated and comprehensive GWAS data become accessible, with the aim of enhancing our knowledge of RA and devising prevention and management strategies for air pollution.

## Supporting information

S1 File (XLSX)

S1 Fig Leave-one-out sensitivity analyses of Mendelian randomization analyses of European populations. (TIF)

S2 Fig Leave-one-out sensitivity analyses of Mendelian randomization analyses of East Asian populations. (TIF)

S3 Fig Scatter plots of Mendelian randomization analyses of European populations. (TIF)

S4 Fig Scatter plots of Mendelian randomization analyses of East Asian populations. (TIF)
