# Supplementary figures and images for: Causal associations of air pollution with rheumatoid arthritis: A transethnic Mendelian randomization study
Source: PLoS One. 2024 Sep 24;19(9):e0307514. doi: 10.1371/journal.pone.0307514 (PMC11421788; doi:10.1371/journal.pone.0307514)

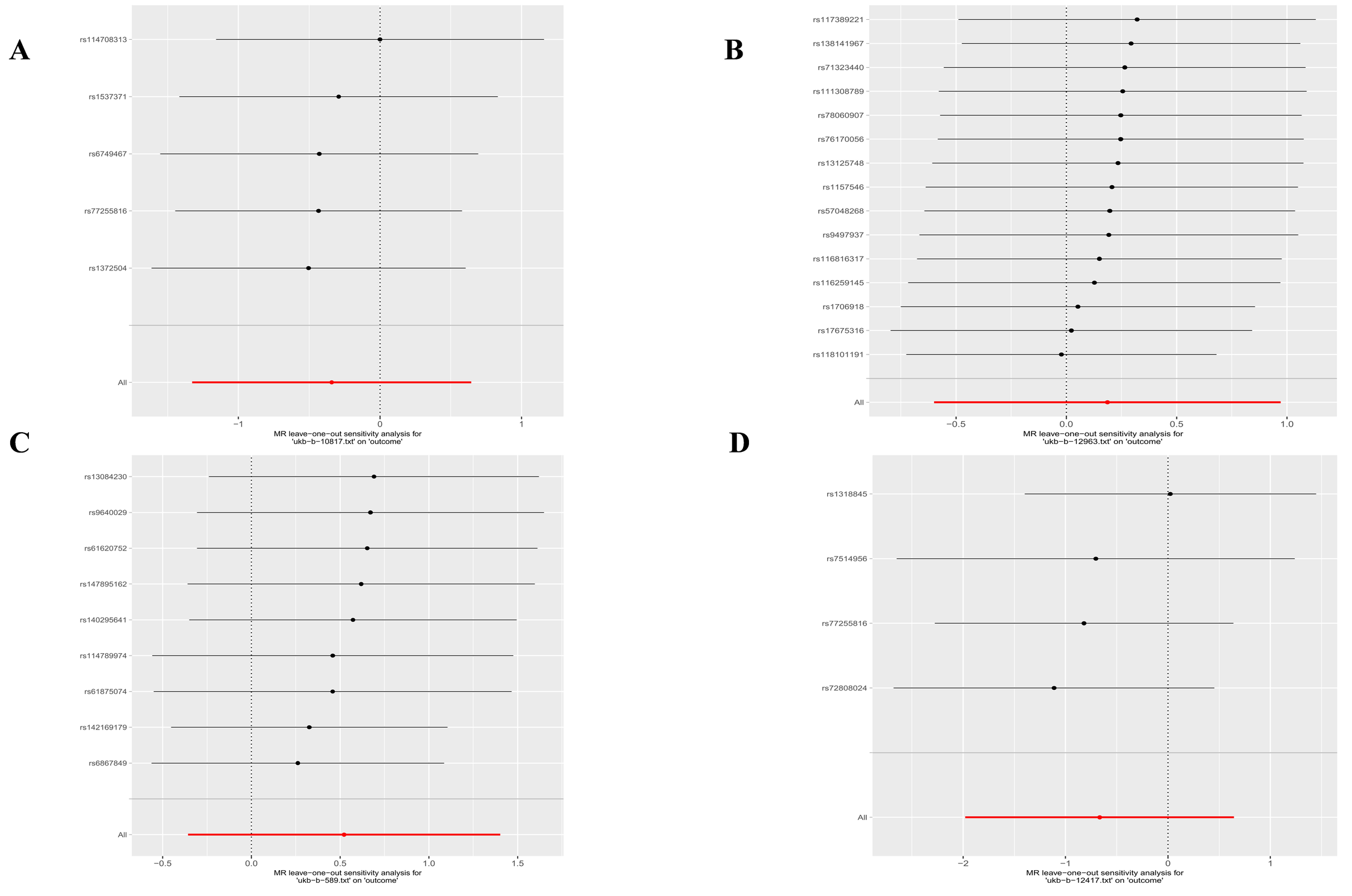

Supplement: S1 Fig — (TIF) [file pone.0307514.s002.tif]

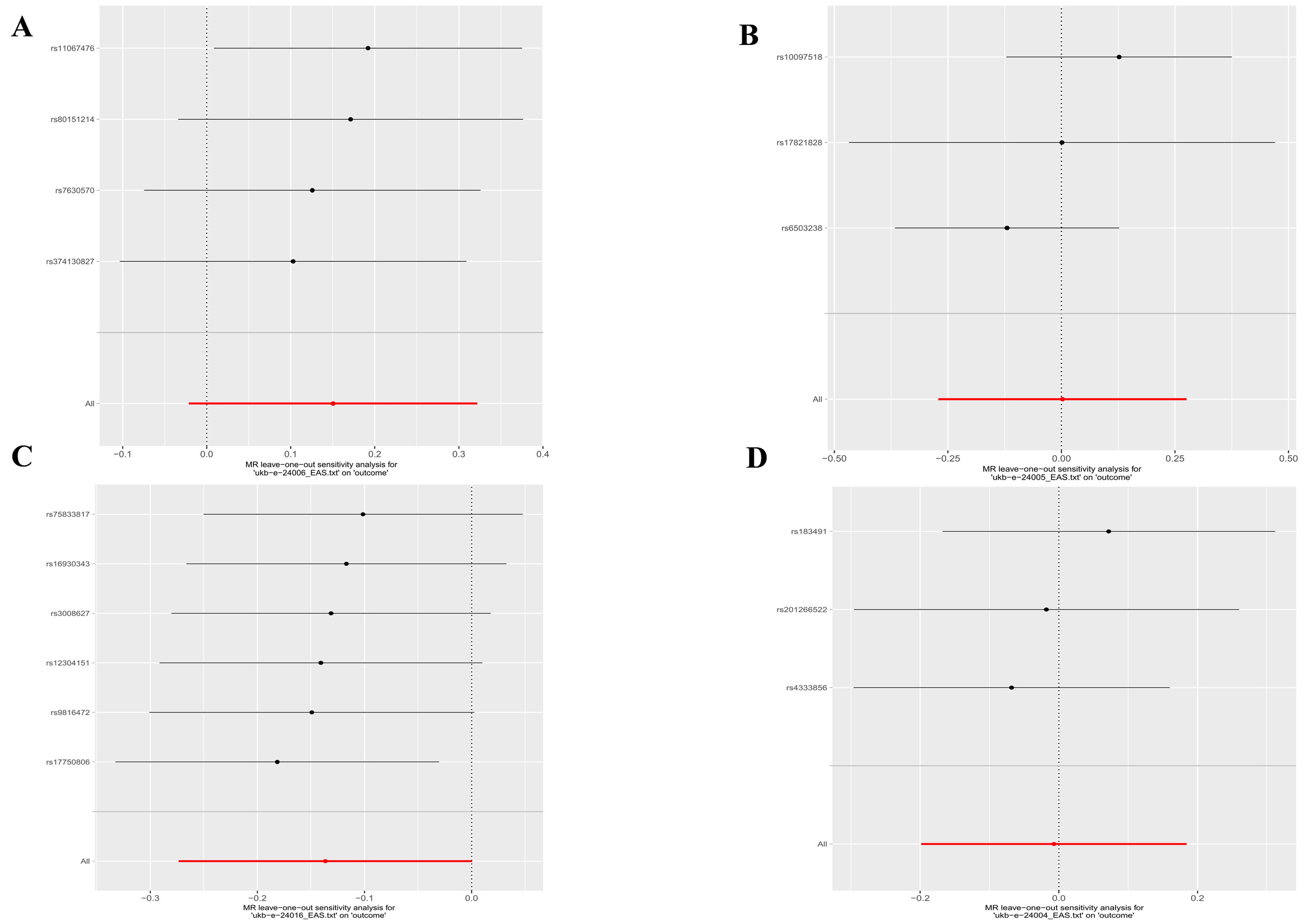

Supplement: S2 Fig — (TIF) [file pone.0307514.s003.tif]

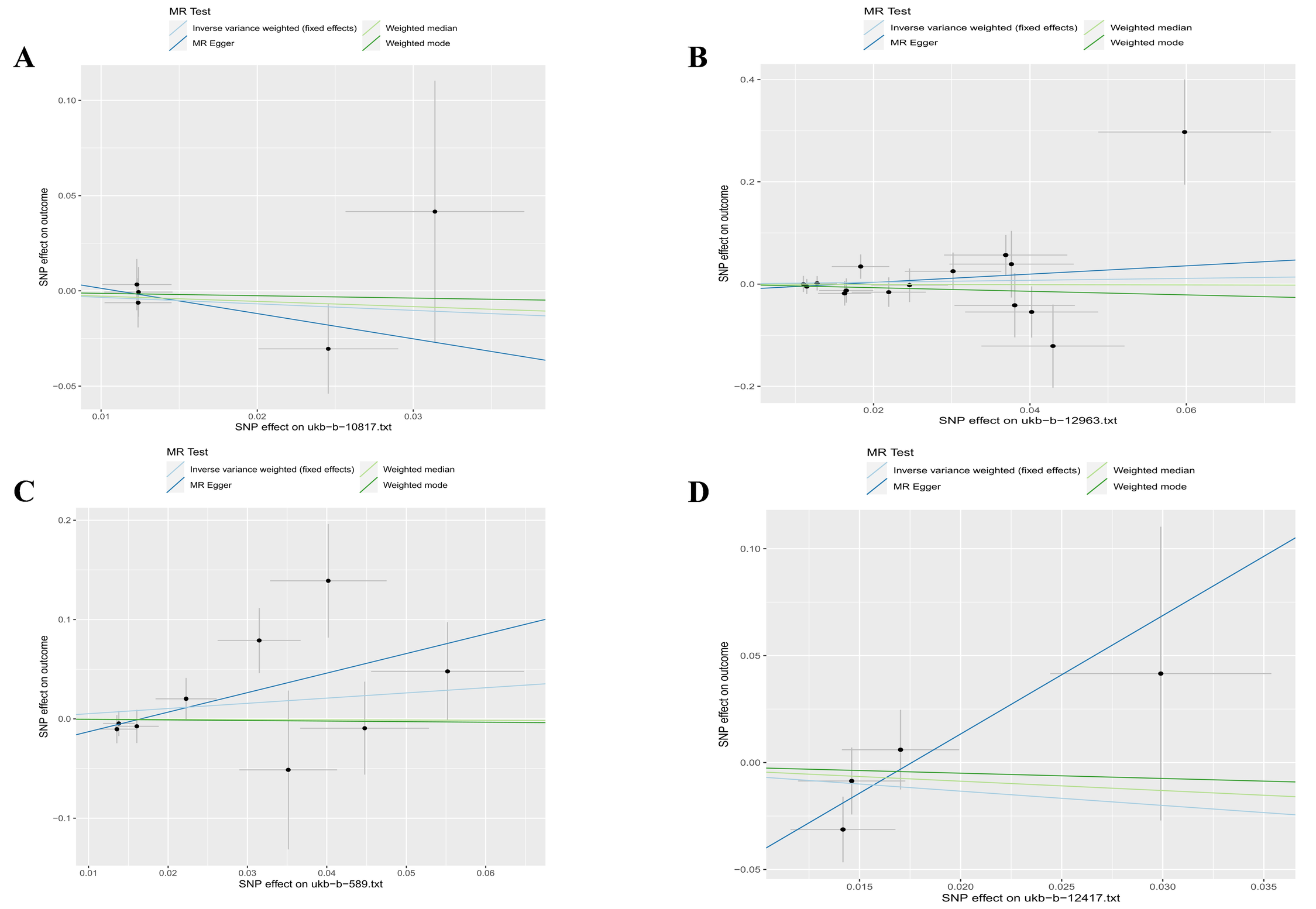

Supplement: S3 Fig — (TIF) [file pone.0307514.s004.tif]

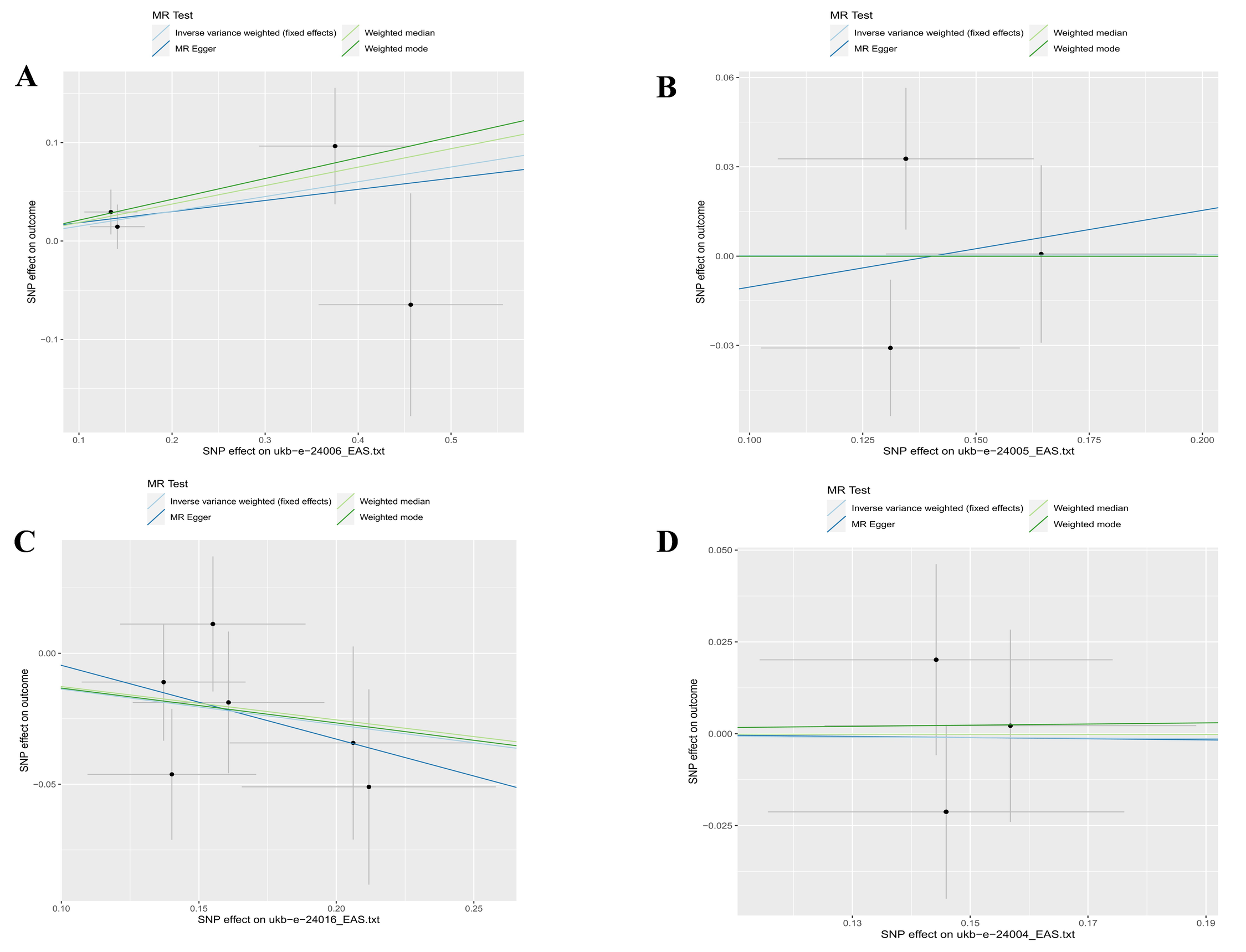

Supplement: S4 Fig — (TIF) [file pone.0307514.s005.tif]
